# Cardiac echo-lab productivity in times of economic austerity

**DOI:** 10.1186/2193-1801-3-703

**Published:** 2014-11-29

**Authors:** Vasiliki K Katsi, Dimitrios A Vrachatis, Anastasia Politi, Manto Papageorgiou, Anastasios Koumoulidis, Ioannis Vlasseros, Manolis Vavuranakis, Dimitrios Tousoulis, Christodoulos Stefanadis, Ioannis Kallikazaros, Kyriakos Souliotis

**Affiliations:** Cardiology Department, Hippokration Hospital, National Health System, Athens, Greece; 1st Department of Cardiology, Hippokration Hospital, National & Kapodistrian University of Athens, Athens, Greece; Department of Statistics, Athens University of Economics and Business, Athens, Greece; Department of Social and Educational Policy, Faculty of Social Sciences, University of Peloponnese, Corinth, Greece; Centre for Health Services Research, Medical School, National & Kapodistrian University of Athens, Athens, Greece

**Keywords:** Health economics, Greece, Echocardiography, Cost-effectiveness

## Abstract

The present study attempts to offer insight into the volume, cost, and productivity of the operation of a cardiac echocardiographic laboratory (echo-lab) in a major public hospital of Greece and thus to contribute, on a practical level, to the widening of knowledge in the strategic field of secondary and tertiary healthcare management. The conducted research includes the basic step of the deployment of a primary data registry in the echo-lab and unfolds in three levels, i.e. the variability measurement of the quantity and cost of medical services provided to different patient populations, the assessment of operating costs and the development of productivity indexes. The results show that the mean costs of provision do change among distinct patient populations. The most important, from a financial standpoint, population cluster appears to be the one corresponding to outpatients. Productivity indices presented in this analysis constitute an essential piece of information which the public healthcare system is currently largely lacking, and which, combined with the pricing and the diagnosis-related group coding system of hospitals, can be used to improve efficiency in the management of secondary and tertiary care.

## Introduction

The recent appropriateness-of-use criteria (Douglas et al. 2011) recognized almost a hundred (out of 202) medical indications in which cardiac echocardiography is considered to have an impact either on diagnosis or in patient management. Therefore, echocardiography laboratories (echo-labs) are an integral department not only of the cardiology clinic in the context of which they operate, but also of the hospital organization as a whole. Similarly, this emerging role of echocardiography contributes in the rise of healthcare costs (Alter et al. 2006; Badano et al. 2009; Levin et al. 2004).

Therefore, health-policy makers have to maintain a challenging equilibrium between (low) budgets vs. capacity constraints of echo-labs, which may result in long patient waiting times (Levin et al. 2004; Freeman 2002; Groene et al. 2008; Groene 2006). For this reason, the organizational, financial and productivity aspects related to the operation of hospital echo-labs have to be assessed, in an effort to improve their cost-effectiveness without impairing quality of care (Badano et al. 2009; Lai et al. 2013).

In Greece, the National Health System (i.e. ESY; established in 1983) has been characterized by problematic administration and low productivity despite a series of legislative reforms (Tountas et al. 2002; Aletras et al. 2007; Halkos and Tzeremes 2011; Kalogeropoulou 2011). During the recent socioeconomic crisis, healthcare system has been pressed to function under significant budget cut-offs. Accordingly, data on the organization, management and implementation of medical and nursing procedures was required by policy-makers in order not to jeopardize quality (Golna and Souliotis 2006).

Based on the above, goal of this study was to assess and understand the existing staffing structure and volume of echocardiographic examinations performed and interpreted in the setting of an echo-lab established in a tertiary hospital. Specifically, the study aimed to (1) determine the annual laboratory volume and types of echocardiographic studies performed, (2) define the average number of studies performed by an echocardiography physician in a year, (3) assess the productivity of echocardiography physicians, and (4) identify factors (programmatic or laboratory related) that affect clinical productivity.

## Methods

### Study population and data collection

In the present observational prospective study a digital registry of (hospitalized or non-hospitalized) patients examined in the echo-lab of a cardiology department of a tertiary hospital in Athens (Greece) was created during a seven-month period (from 10/4/2010 to 26/11/2010).

Information regarding echocardiographic study and patient characteristics was recorded as following: (i) type of the echocardiographic study (2D-ECHO; TEE; 3D-ECHO; STRESS-ECHO), (ii) costing of the echocardiographic study (costed vs. non-costed), (iii) financial value of the echocardiographic study, (iv) type of referral (outpatients with a NHS referral; outpatients with a freelancer referral; inpatients) (v) repetitiveness of the examination (initial contact; follow-up – studies were classified as “follow-up” if only the patient has already been examined in our laboratory at least once), (vi) patient age, (vii) patient gender, (viii) patient place of residence (Prefecture of Attica; other) and (ix) patient insurance capacity (social insurance; private insurance; no insurance).

The data collected from the register were properly encrypted for confidentiality reasons.

### Calculation of costs

Wage costs and variable echocardiography test costs were estimated in the study. Fixed cost (including capital, depreciation and service cost), as well as induced costs reflecting the additional cost that arises due to complications of testing were not taken into account, for simplicity reasons.

The echo-lab operated on a 7-hour, 5-day basis. Only one echocardiography system was in use, and therefore only one patient could be examined at a time. As physicians had other clinical responsibilities to attend to in addition to echocardiography coverage, about 1/3 of their estimated regular working hours was appointed to this field. Echocardiography services were also provided by a doctoral candidate, who was not on the hospital’s payroll. The estimation of aggregate wage costs was based on the salary scales in accordance with the provisions of law. Monthly wage costs are presented in Table 1.

**Table 1:** **Monthly wages**

Grade	Monthly salary in euros (€)
Director	2,043
SpR1	1,659
SpR2	1,320
PhD student	0
SMW	5,022

SpR1 Specialist Registrar 1:, SpR2: Specialist Registrar 2, SMW: sum of monthly wages.

Source: Government gazette A297 “Wage setting for public officials, the police, the fire brigade and the port police and other relevant provisions” issued on 30-12-2003.

For the calculation of total wage costs the total man-hours produced in the lab were taken into account. Calculations are based on the annual number of days of insurance, which amount to 300 days (25 days per month). In particular, the number of insurance days of the study period equals to 189.17 days, as shown below:

- From 10/4/2010 to 30/4/2010: 21 calendar days, or 17.5 days of insurance
- From 01/5/2010 to 31/10/2010: 6 months, or 150 days of insurance
- From 01/11/2010 to 26/11/2010: 26 calendar days, or 21.67 days of insurance

The calculation of total wage costs for the study period is approached according to the following formula:

Where*:*

*TWC*, is the total wage cost in the reference period

*SMW*, is the sum of monthly wages of the lab’s staff, calculated as *SMW* = *€5,022*

*m*, is the number of months in the reference period, calculated as *m = 6 + 21/30 + 26/30 = 7.567*

*h*, is an adjustment factor to the actual daily work hours in the lab, set at *h = 1/3*

In addition, the total cost of echocardiographic studies (variable cost) performed was calculated based on the volume of studies performed and the price of each type of study. Details on the prices of echocardiography procedures applied in the study calculations are presented in Table 2.

**Table 2:** **Price list of echocardiographic studies**

	Type of echocardiographic study			
Value	Costed, €		Non Costed, €	
	2D-ECHO	TEE	3D-ECHO	STRESS-ECHO
Social Insurance Fee	58	85	-	-
Market Price	-	-	-	190*

2D (3D): two (three) dimensional; ECHO: echocardiograpy; TEE: transesophageal.

Source: Government gazette B3100 “Costing of medical procedures and tests” issued on 30-11-2011.

*Based on the affiliated hospital’s call.

### Statistical analysis

Sample characteristics are presented in Table 3. Measures of central tendency (arithmetic mean) and dispersion (standard deviation) are used in order to measure quantitative variables. Age, gender and place of residence are examined as possible risk factors of demand intensity and cost. Interactions between risk factors are investigated with factorial designs. The interdependence between quantitative variables is investigated with the Pearson’s coefficient of correlation (r). Odds ratios are also used to measure risk factor effects. The level of statistical significance is set at the 1% level.

**Table 3:** **Sample characteristics**

*Population characteristic*	N	%	Age (SD)
***Gender***			
Males	606	60.9	62.8 (5.2)
Females	389	39.1	62.8 (5.2)
***Place of residence***			
Attica	708	71.2	62.9 (16.2)
Outside Attica	287	28.8	63.8 (16.0)
***Insurance coverage***			
Social security	973	97.8	63.2 (16.1)
Private insurance	1	0.1	52.0
Uninsured	21	2.1	58.7 (16.1)
***Referral type***			
Outpatients	635	63.8	60.6 (16.6)
*NHS referral*	*563*	*56.6*	*60.4 (16.5)*
*Freelancer referral*	*72*	*7.2*	*62.2 (17.2)*
Inpatients	360	36.2	67.6 (14.3)
***Costing type***			
Costed	796	80.0	62.6 (12.9)
*2D-ECHO*	*753*	*75.7*	*62.9 (16.7)*
*TEE*	*43*	*4.3*	*56.8 (16.9)*
Non-costed	199	20.0	63.2 (16.9)
*3D-ECHO*	*77*	7.8	60.7 (18.0)
*STRESS-ECHO*	*122*	12.2	63.3 (9.6)
***Repetitiveness***			
Follow-up	259	26.0	64.0 (13.9)
Non-follow up	736	74.0	62.7 (16.9)
***Total***	***995***	***100.0***	***63.1 (16.1)***

SD: Standard Deviation.

With regards to the estimation of productivity parameters, these were assessed according to the following indices: (a) number of studies per physician (NSP), defined as the total number of studies in the surveyed period divided by the total number of physicians (excluding the doctoral candidate), (b) number of studies per physician per day (NSPD), defined as the NSP divided by the number of insurance days.

### Ethics, financial issues and patient consent

Patients were not exposed at any additional risks associated with their participation in the study. No extra costs, except from those related with the routine echo-lab operation were induced by the conduct of the study. Echocardiographic studies utilized for patient evaluation were selected upon consensus of the referring physicians and the cardiologists in charge of the echo-lab according to respective medical indications. The study conforms to the principles outlined in the Declaration of Helsinki (World Medical Association Declaration of Helsinki 2013). The institutional review board (Hippokration Hospital, Athens, Greece) approved study protocol. All patients provided informed consent authorizing researchers for the collection, analysis and release of their medical information.

## Results

### Utilization and cost of services

The echocardiographic studies performed in the echo-lab cost €73,271. Total wage costs (TWC) equaled €12,667 increasing the total cost of the provided health services to €85,938, which annualized is equal to €136,287 (=85,938∗300/189.17).

Table 4 cross classifies total costs according to the referral type of patients and the costing type of studies. The health services provided toward outpatients cost approximately €61,593 (=€51,459 + 0.8*12,667), namely 71% of the echo-lab’s total operating cost.

**Table 4:** **Descriptive measures of the cost of cardiac echocardiographic studies by costing type of echocardiography and type of hospitalization**

	***Costing type of echocardiographic studies***											
	Costed studies				Non-costed studies				Total			
***Hospitalization type***	Sum €	%	Mean €	SD €	Sum €	%	Mean €	SD €	Sum €	%	Mean €	SD €
**Outpatients**	**28,044**	**54.5**	**58.8**	**4.6**	**23,415**	**45.5**	**159.9**	**54.7**	**51,459**	**100.0**	**81.0**	**54.6**
*NHS referral*	*25,148*	*56.7*	*58.8*	*4.5*	*19,235*	*43.3*	*142.5*	*80.6*	*44,383*	*100.0*	*78.8*	*53.3*
*Freelancer referral*	*2,896*	*40.9*	*59.1*	*5.4*	*4,180*	*59.1*	*181.7*	*39.6*	*7,076*	*100.0*	*98.3*	*61.8*
**Inpatients**	**18,772**	**86.1**	**58.9**	**4.7**	**3,040**	**13.9**	**74.2**	**93.8**	**21,812**	**100.0**	**60.6**	**32.0**
***Total***	***46,816***	***63.9***	***58.8***	***4.6***	***26,455***	***36.1***	***132.9***	***86.0***	***73,271***	***100.0***	***73.6***	***48.7***

SD: Standard Deviation.

The vast majority (98%) of the patients had insurance coverage. The remaining 2% (21 patients) were provided with cardiac echocardiographic studies that cost €1,261.

Age or residence of the patients did not interact with the costing type of studies (p-values 0.31 and 0.94 respectively) and were further evaluated as not significant risk factors of the echo-lab’s operating cost (p-values 0.553 and 0.07, respectively). On the contrary, the gender of patients had a significant effect on the cost distribution, yet only within the outpatient population (p-value 0.007), where as seen, male outpatients produced higher mean costs (€85.4 (SD €57.7)) than female ones (€73.8 (SD €48.5)). Repetitiveness of studies did not interact with age (p-value 0.313), gender (p-value 0.045) or place of residence of the patients (p-value 0.629).

As Table 4 shows, the general mean cost was estimated at €73.6 (SD €48.7). The mean cost differed noticeably between the two levels of the costing factor, being estimated at €58.8 (SD €4.6) in the group of costed studies and at €132.9 (SD €86.0) in the group of non-costed studies. Indeed, the differential effects of the costing type on the cost of echocardiographic studies were found significant (p-value < 0.001). It was further observed that higher rates of the STRESS-ECHO category in particular, generated higher costs. This was the case of outpatient males, outpatients with freelancer referrals and patients who reside in Attica.

The referral type interacted significantly with the costing type (p-value < 0.001). Outpatients performed more non-costed studies than inpatients (OR 2.58, CI: (1.58, 4.20)) and eventually had significantly higher mean costs (p-value < 0.001). Follow-ups and follow-ups of non-costed studies were also more frequent in the outpatient group compared to the inpatient one. The OR of followups between outpatients and inpatients was 2.87 (CI: (1.84, 4.46)) whereas the OR of followups of non-costed studies between outpatients and inpatients was 1.59 (CI: (1.02, 2.46)).

On a study type level, outpatients with freelancer referrals performed STRESS-ECHO studies at a higher rate compared to outpatients with NHS referrals and inpatients (43.1% vs. 24.3% vs. 6.1%). Outpatients with freelancer referrals represented the group with the higher mean costs (€98.3, SD €61.8). The mean cost for the three categories was €98.3 (SD €61.8), €78.8 (SD €53.3) and €60.6 (SD €32.0), respectively (Table 4). The proportion of STRESS-ECHO studies was higher in the group of patients residing in Attica compared to patients living outside Attica (20.7% vs. 15.3%). The mean cost of the two groups were estimated at €75.4 (SD €49.6) and €69.2 (SD €46.0), respectively. Finally, male outpatients carried STRESS-ECHO studies at the rate of 31.2% and at mean cost €85.4, while the corresponding estimates for female outpatients were 18.7% and €73.8 respectively.

With regards to the productivity indices, the NSP index for the reference period was estimated at (NSP = 995/3) 332 studies/physician, while the NSPD was equal to (NSPD = 995/189.17) 5.26 studies/physician/day. In annual terms, the number of studies and the NSP index were (annual number of echocardiographic studies = 5.26*300) estimated at 1578 and (annual NSP = 1578/3) 526 respectively.

## Discussion

Advances in cardiac technology and expansion of its clinical application, combined with the rise in the prevalence of cardiovascular disease have increased the utilization of cardiac technologies and the associated healthcare costs, having a significant contribution to total healthcare expenditure growth (Alter et al. 2006; Levin et al. 2004). In the US, between 1993 and 2001, there was an almost 3-fold increase in the use of imaging stress tests (Levin et al. 2004), while between 1999 and 2004 echocardiography services grew by c. 8% per year (Pearlman et al. 2007). In Canada, the annual expenditure associated with the use of cardiac technology incurred a 2-fold increase between 1992 and 2002, with echocardiography test costs exceeding CAN$498 million over the 10-year period (Alter et al. 2006).

In the era of cost-containment and limited healthcare budgets, the need to enhance healthcare delivery efficiency, optimize costs and improve hospital performance is becoming a priority for healthcare systems, especially in countries like Greece, in which the NHS has been long characterized by inefficiencies, poor management and low productivity (Economou 2010). Yet, cost assessment and productivity evaluation of healthcare units in the Greek NHS is still a goal to be set. In fact, a few years ago the cost of the services provided by NHS to social security funds (on behalf of the patients) was estimated as a product of hospitalization days per se. Recent NHS reform has implemented obligatory pricing of the hospital healthcare services through a coding system for diagnosis-related groups. Subsequently, cost analysis in terms of a procedure coding system is now feasible but is only being conducted unofficially in very few centers due to individual initiative. Therefore, to the best of our knowledge there are no available data of physician productivity, especially in the field of cardiac echocardiography laboratories.

In this dynamic field, the present study, attempting to gain a better perspective on the volume, cost, and productivity triptych of the operation of a public hospital’s cardiac echocardiographic laboratory was conducted on a triple level applying a novel primary data register. Firstly it provided with a combination of descriptive measures of the quantity of medical services provided in the lab, across different target patient populations. Secondly it estimated the total cost of the provided echocardiographic studies confirming that the mean costs of provisions do change among the distinct populations of users. Thirdly, it explicitly proposed ways of measuring the echo-lab’s productivity of labor and offered original values for the estimation of the parameters that were estimated in this direction.

Higher study costs were observed in the outpatient group, which appears to incline to the utilization of non-costed echocardiographic studies. Taking this finding a step further, it was observed that outpatients that are men or have a freelancer referral are more strongly correlated to the costlier non-costed ‘portfolio’ of healthcare services of the echo-lab under analysis.

Finally, the study calculated the number of echocardiographic studies per physician and the number of studies per physician per day, as a means of assessing productivity. Productivity indices presented in this analysis constitute an essential piece of information which the public healthcare system is currently largely lacking, and which could be used to make comparisons, detect inefficiencies and capacity constraints, and thus improve the performance of the cardiology clinics of the Greek NHS.

In the context of the comprehensive reform that the Greek health system and is undergoing and the general need to increase efficiency in the public sector, it is important for policy-makers, hospital managers and also physicians performing echocardiography tests to comprehend the relationship between the cost and the effectiveness of tests, including how to measure each of these parameters, as well as the importance of their appropriate use, based on research evidence. The present study attempted to provide an estimate on direct echocardiography lab costs in the Greek NHS, not accounting for fixed costs or indirect costs associated with the complications of testing, and not assessing appropriateness of use, i.e. whether the tests performed met appropriate use criteria. However, as recording of such information is not a routine policy in our institution, the study duration was limited in a 7-month period. Given that, we believe the present study provides original data in this field regarding our country which could potentially be of use for health policy makers. Undoubtedly, further studies are needed for a full assessment of costs and effectiveness of cardiac echo-labs operating in the Greek NHS.
